# The Role of Intervention Fidelity, Culture, and Individual-Level Factors on Health-Related Outcomes Among Hispanic Adolescents with Unhealthy Weight: Findings from a Longitudinal Intervention Trial

**DOI:** 10.1007/s11121-023-01527-z

**Published:** 2023-04-18

**Authors:** Padideh Lovan, Alyssa Lozano, Yannine Estrada, Cynthia Lebron, Tae Kyoung Lee, Sarah E. Messiah, Guillermo Prado

**Affiliations:** 1 Department of Psychology, University of Miami, Miami, FL USA; 2 Sylvester Comprehensive Cancer Center, University of Miami, Miller School of Medicine, Miami, FL USA; 3 Department of Public Health Sciences, University of Miami Miller School of Medicine, Miami, FL USA; 4 School of Nursing and Health Studies, University of Miami, Miami, FL USA; 5 Department of Child Psychology and Education, Sungkyunkwan University, Seoul, South Korea; 6 Department of Epidemiology, Human Genetics and Environmental Science, The University of Texas Health Science Center at Houston, School of Public Health, Dallas, TX USA; 7 Center for Pediatric Population Health, University of Texas Health Science Center, School of Public Health, Dallas, TX USA; 8 Department of Pediatrics, McGovern Medical School, Houston, TX USA

**Keywords:** Fidelity, Culture, Health outcomes, Hispanic youth, Parenting strategies, Family intervention

## Abstract

Previous studies have suggested the impact of intervention fidelity on the management and prevention of chronic diseases; however, little is known about the effect of the contributing determinants (at multiple levels of influence) that can impact health-related interventions intending to improve the health status of Hispanic adolescents with overweight or obesity. The current study aimed to assess whether fidelity (i.e., dosage and quality of the program delivery), acculturation (i.e., orientation to the American culture, retention of Hispanic cultural values), and individual-level socio-demographic characteristics (i.e., income, education) predict changes in family processes (e.g., parent control), which in turn may affect adolescent health-related outcomes including body mass index (BMI), physical activity, dietary intake, and adolescents’ health-related quality of life. A pathway analysis model was utilized to explore the study variables among 140 Hispanic parent-adolescent dyads randomized to Familias Unidas Health and Wellness (FUHW) intervention. Results indicated that fidelity was significantly associated with changes in parent-adolescent communication, parent monitoring, limit-setting, and control. Parents’ education was associated with changes in parent limit-setting, and parent Hispanicism was associated with changes in parent limit-setting and discipline. The examination between family processes and adolescent health outcomes revealed that parents’ higher discipline and improved communication with their adolescents were significantly associated with improved adolescents’ quality of life, and parent control was positively associated with physical activity and negatively associated with BMI in adolescents. Our findings demonstrated the significant contribution of intervention fidelity and participants’ characteristics in parenting strategies leading to adolescents’ health outcomes to prevent obesity-related chronic diseases. Future research is needed to investigate the effect of environmental and organizational factors on the delivery of the intervention materials.

Family-based preventive interventions have demonstrated efficacy and effectiveness in the prevention and reduction of poor health outcomes among youth (Sandler et al., 2015), yet these interventions are not widely available to the community. Of particular importance is the delivery of evidence-based interventions (EBIs) with fidelity, the degree to which an intervention is delivered as intended by the program developers (Carroll et al., 2007). However, limited studies have explored the association between fidelity and health-related study outcomes longitudinally, particularly among ethnic minorities or adolescents with overweight or obesity. Therefore, this study will explore determinants at multiple levels of influence that can impact health-related outcomes in interventions intending to improve the health status of Hispanic adolescents with overweight and obesity. Understanding these determinants is particularly important because Hispanic adolescents are at a higher risk of being overweight and obese, compared to non-Hispanic youth, in turn putting them at risk for cardiovascular disease, cancer, and other chronic diseases during adulthood. Specifically, we will examine fidelity, socio-demographic characteristics, and acculturation as predictors of adolescent health-related outcomes [i.e., quality of life, body mass index (BMI), physical activity (PA), and dietary intake] through the mediating role of family processes including parent-adolescent communication, parent monitoring, parent-limit setting, parent control, and parent discipline.

## Intervention Fidelity

Fidelity is critically important to measure and achieve when translating interventions to clinical and community practice because poor fidelity can lead to negative health outcomes (Cohen et al., 2008). Moreover, as preventive interventions are being disseminated in a diverse population, fidelity may improve positive outcomes (St George et al., 2016). This study will evaluate two core dimensions of fidelity: (1) “dosage” or the amount of program delivery; and (2) the quality of program delivery (Dane & Schneider, 1998).

Previous research has operationalized dosage as participant attendance, an important factor in determining intervention efficacy (Charlebois et al., 2004). Evidence suggests that higher program dosage is associated with better intervention outcomes (Smokowski et al., 2016). However, a limited number of studies have examined the long-term effect of intervention dosage on participants’ outcomes. Another study demonstrated a positive relationship between parents’ attendance and improvement in their discipline as part of their parenting strategies, leading to improved study outcomes in the youth (Prado et al., 2006). Additionally, Jeffers et al. (2019) suggested that higher participants’ attendance in a diabetes prevention program is related to increased patient activation and greater expression of committing to healthy behaviors at 4 months. However, further evidence is required to generalize the mentioned findings to family-based obesity preventive interventions with Hispanics. Another important dimension of fidelity to measure is the quality of program delivery because the poor quality of program delivery diminishes the effects of interventions (Carroll et al., 2007). For example, Hahn and colleagues found that poor implementation of life skills training (LST), an intervention designed to be implemented in middle schools, had diminished effects when it was not implemented with high fidelity (Hahn et al., 2002). In summary, both optimal program dosage and quality of program delivery are critically important to achieve when translating evidence-based interventions to practice.

The current study is grounded in the ecodevelopmental theory (Coatsworth et al., 2002) for obesity. Ecodevelopmental theory is a theoretical framework utilized to explain the relationships between risk and protective processes associated with adolescents’ health-related outcomes by focusing on the influence of multiple social-ecological levels. We used this theory to investigate the impact of multiple levels of influence including intervention fidelity, socio-demographic characteristics, acculturation, and family-level processes on adolescents’ health-related outcomes. Below, we describe the variables used in this study along with the impacts reported by previous findings.

## Socio-Demographic Characteristics and Acculturation

Parent socio-demographic characteristics (e.g., income and educational level) may also affect adolescents’ health-related outcomes. For example, lower family income and lower parents’ education levels are associated with a higher risk of obesity, lower fitness levels, and lower quality of life among adolescents (Huang et al., 2021). In addition to socio-demographic characteristics, acculturation may also impact adolescents’ health status. Acculturation is known as a multidimensional process, where the traditional cultural values of one’s home country and the cultural values of the host country merge during the process of immigration (Schwartz et al., 2010). It is thought that some of the aspects of the Hispanic culture are protective and its deprivation through adaptation to a new culture (e.g., lower Hispanicism) may cause negative health outcomes, which explains the “Hispanic Paradox” (Llabre, 2021). A great example for the Hispanic Paradox phenomenon is heart disease. Despite presenting higher cardiovascular risk factors, Hispanics often face a lower burden of heart disease compared to their non-Hispanic counterparts, which may be related to social connections that function as a protective cultural aspect, which results in less stress accumulation in this population (Llabre, 2021). Additionally, parents’ acculturation levels may affect their parenting strategies as higher Hispanicism is suggested to cause higher directive food parenting practices such as limiting less-healthy foods (Power et al., 2015).

## Family-Level Processes

In addition to sociodemographic characteristics and acculturation, family-level processes play a critical role in healthy adolescent development (Viner et al., 2012). Family is particularly influential for Hispanic adolescents due to *familismo*, a cultural value that emphasizes the importance of family harmony (Unger & Schwartz, 2012). In prior studies, family processes such as parent–adolescent communication have been found to be related to PA and dietary intake (Prado et al., 2020). Family communication quality (e.g., open versus closed communication) also impacts multiple factors of adolescent developmental outcomes. Previous findings indicated that open family communication creates a positive family environment, where adolescents can express their feelings, show fewer depressive symptoms, and report higher satisfaction in life, whereas problems in family communication may lead to conflict and misunderstanding (Ioffe et al., 2020).

Additionally, family-level processes that are related to parenting strategies for dietary and physical activity-related behaviors are also associated with PA, dietary intake, and BMI (LeCroy et al., 2019). There are several different strategies (e.g., disciplining and monitoring) that can be used to assess how parents manage the intake of snacks and/or sugar-sweetened beverages as well as sedentary behaviors (Larios et al., 2009). Results from a review study related to parenting strategies and youth health-related outcomes suggested that improved parenting and child management skills (e.g., higher monitoring, discipline, and reinforcement) lead to positive behaviors (e.g., quality diet and higher physical activity) and better weight loss (Kitzman-Ulrich et al., 2010). Taken together, family-level processes, whether related to general family functioning (e.g., parent-adolescent communication) or specific to obesogenic behaviors (e.g., parent discipline), may directly or indirectly impact adolescent health-related behaviors. However, limited data are available regarding the mentioned relationships in ethnic minority populations, particularly Hispanics.

The current study used data from a study designed to assess the efficacy of an adaptation (St George et al., 2018) of Familias Unidas (Prado & Pantin, 2011) for the prevention and reduction of obesity-related behaviors in Hispanic youth and their parents. This study aimed to assess whether fidelity (i.e., attendance and quality of program delivery), acculturation (i.e., Americanism and Hispanicism), and socio-demographic characteristics (i.e., income, education) predict family processes (e.g., parent-adolescent communication) and adolescent health-related outcomes (e.g., physical activity). The following relationships were hypothesized: (a) fidelity will be positively associated with changes in family processes; (b) socio-demographic characteristics and acculturation will be positively associated with changes in family processes; (c) changes in family processes will be positively associated with adolescents’ physical activity and dietary intake and negatively associated with adolescents’ BMI and quality of life (number of bad days); (d) fidelity, socio-demographic characteristics, and acculturation will be positively associated with adolescents’ physical activity and dietary intake and negatively associated with adolescents’ BMI and quality of life (number of bad days) with changes in family processes having a mediating effect.

## Methods

### Participants

Participants were 140 Hispanic adolescents with overweight and/or obesity and their parent/primary caregiver, who were recruited from middle schools in Miami-Dade County, Florida and randomized to the experimental condition of the study, Familias Unidas for Health and Wellness (FUHW) (Prado et al., 2020; St. George et al., 2018). Recruitment took place in 2015 by the study staff. Youth were eligible to participate if they: (1) identified as Hispanic, (2) were in the 7th or 8th grade, (3) lived with an adult primary caregiver who was willing to participate in the study, (4) planned to stay in South Florida for the duration of the study, and (5) had a BMI of ≥ 85th percentile adjusted for age and sex (Centers for Disease Control & Prevention, 2021; Kuczmarski et al., 2002). Exclusion criteria were (1) adolescents with a BMI of < 85th percentile adjusted for age and sex, (2) if either parents or adolescents had a serious health issue (e.g., heart problems) and they were not able to complete the PA tests. Participants received incentives ($50–$85) for completing each stage of the study assessment. The study CONSORT is published elsewhere (Prado et al., 2020). Table 1 presents details regarding participants’ demographic characteristics.

**Table 1:** Participants characteristics at baseline

**Characteristics**	***n*/Mean (SD/%)**
**Adolescents**	
Age, year	13.03 (0.86)
Sex	
Male	71 (50.7%)
Female	69 (49.3%)
Nativity status	
US born	85 (60.7%)
Foreign born	55 (39.3%)
BMI percentile categories	
Overweight	57 (40.7%)
Obese	61 (43.6%)
Severely obese	19 (13.6%)
**Parents**	
Age, year	42.09 (6.30)
Sex	
Male	18 (12.9%)
Female	122 (87.1%)
Nativity Status	
US born	14 (10.0%)
Foreign born	126 (90.0%)
BMI categories	
Normal weight	18 (12.9%)
Overweight	49 (35.0%)
Obese	72 (51.4%)
Household annual income, US $	
< 30,000	84 (60.0%)
30,000–49,999	29 (20.7%)
> 50,000	21 (15.0%)
Parents’ education	
No education	2 (1.4%)
Elementary	5 (3.5%)
High school	66 (47.2%)
College	55 (39.2%)
Grad school	12 (8.6%)

BMI percentile categories: overweight = 85th–95th percentile, obese = 95th–98th percentile, severely obese > 99th percentile; body mass index categories: normal weight: BMI = 18.5–24.9, overweight: BMI = 25–29.9, obesity: BMI ≥ 30

### Procedures

The data from this study comes from a randomized controlled trial evaluating the efficacy of a family-centered intervention, FUHW, relative to a control condition, in reducing BMI, increasing physical activity levels, and improving diet quality. The study was approved by the University of Miami’s Institutional Review Board (IRB) and by the Miami-Dade County Public School System’s IRB. FUHW is a 12-week intervention consisting of eight parent group sessions (2.5 h each) and four individual family sessions (1 h each). During the group sessions, the first 1.5 h included discussions about various topics (e.g., open family communication) and role-playing between the parents led by two trained bilingual facilitators while adolescents were engaged in an outdoor physical activity led by trained local park coaches. During the second hour of group sessions, both parents and adolescents were involved in hands-on nutrition education and training activities such as cooking and recipe preparation. Furthermore, the group sessions allowed the families to exchange information and connect regarding family and cultural values through group discussions that covered various topics such as how cultural norms in their country of origin are different than the ones in the USA. During the family sessions, facilitators met with the families individually to practice the skills the parents learned during the group sessions with their adolescents (e.g., role-playing related to adolescent’s lifestyle). To promote attendance, our first family session is partially designed to address barriers and facilitators to program attendance. Facilitators also contacted the participants in advance to remind them to attend the upcoming session. To improve the quality of program delivery, all facilitators were trained before the intervention starts, facilitators were supervised on a weekly basis, and all the sessions were recorded to conduct fidelity ratings, which were discussed later with the facilitators by the clinical supervisors. Details regarding the intervention activities and rationale are published elsewhere (St George et al., 2018). Data were collected at baseline (time 1), 6 months (time 2), 12 months (time 3), and 24 months (time 4) post-baseline and measures were offered in both English and Spanish.

### Measures

#### Fidelity

##### Dosage

Attendance at the intervention sessions was used to measure dosage. Initial attendance was calculated by summing attendance across the first three sessions. Continued attendance was measured by summing attendance at the remaining (i.e., sessions four to 12) sessions. We decided to include the first three intervention sessions (i.e., one family session and two group sessions) in the initial attendance variable since the theme of all three sessions were similar with the aim of increasing participants’ engagement containing intervention orientation, goal setting, and identifying communication barriers and facilitators. This approach is also consistent with our prior work (Prado et al., 2006).

##### Quality of Program Delivery

The quality of program delivery was measured using session-specific forms for group sessions and family sessions adapted from our past studies to examine the degree to which the intervention was delivered as intended by the trained facilitators (St. George et al., 2018). Independent master’s level graduate student raters, trained to criteria, randomly selected 25% of intervention sessions to rate on four dimensions. The criteria for the group session ratings included: joins all members, established group member alliances, utilizes problem-posing and participatory learning methods, and acts as a switchboard and/or speaks for a long period of the activities. The criteria for rating the family sessions included: joins all family members and acts as a switchboard and/or speaks for a long period of the activities. Raters used a 7-point Likert scale with 0 indicating the lowest quality of program delivery and 7 indicating the highest quality of program delivery. To check inter-rater reliability, 20% of the rated sessions were coded by another rater independently (*κ* = 0.41, *p* < 0.001). To be consistent with our previous studies (St. George et al., 2016), we used the average of overall session quality scores for the analyses. The FUHW facilitator manual highlights several “key points” that should be made in each session. For example, a key message for the facilitators to stress in the first parent-adolescent group activity is to explain and empower families to make healthy food choices (e.g., eating more fruits and vegetables). This message is instilled in parents throughout the intervention through other opportunities to implement these learned skills.

#### Acculturation

##### The Bicultural Involvement Questionnaire

The bicultural involvement questionnaire (Szapocznik et al., 1980) was used to assess parents’ acculturation orientation to the American culture and retention of Hispanic cultural values among parents. Reliability and validity have been established, for both content and construct, for use of this measure with a Hispanic sample (Coatsworth et al., 2002) and included 22 items with two subscales (Americanism and Hispanicism, 11 items each), which assessed the degree of participants’ comfortability with both cultures within multiple settings such as work, home, or social circles. Responses use a 5-point Likert scale from 1 representing “Not at all comfortable” to 5 representing “Very Comfortable.” An example item was “*How comfortable do you feel speaking English at home*?” For this study, Cronbach’s *α* = 0.84 and Cronbach’s *α* = 0.95 for Hispanicism and Americanism, respectively.

#### Family Processes

##### Parent-Adolescent Communication Scale

Parents were asked to complete the 20-item parent-adolescent communication scale (Barnes & Olson, 1985) to assess the quality of open and closed communication with their adolescents. Open family communication measures the degree to which parents and adolescents can communicate freely and express their opinions. Closed family communication measures the obstacles in family communication such as reluctance to disclose opinions. Response options were on a 5-point Likert scale ranging from “*strongly disagree*” to “*strongly agree*.” An example item includes: “*I am very satisfied with how my child and I talk together.*” Cronbach’s *α* = 0.91. This measure was modified for diverse ethnic minority groups, particularly Hispanics, and has been used in numerous all-Hispanic studies (Estrada et al., 2017; Prado et al., 2012; Pantin et al., 2003).

##### The Parenting Strategies for Eating and Activity Scale

The parenting strategies for eating and activity scale, which consists of 26 items and 5 subscales (Larios et al., 2009), was used to measure health-related parenting strategy patterns. Constructs assessed included limit setting (6 items, measured the frequency of restrictive approaches used by parents to limit unhealthy eating and physical inactivity), monitoring (7 items, measured the frequency of parents monitoring on youths’ healthy behaviors), discipline (5 items, measured the frequency of disciplinary practices to limit unhealthy eating and physical inactivity), control (6 items, measured the control techniques used by parents), and reinforcement (2 items, measured the parents’ use of praise when their children are physically active and/or had a healthy meal/snack; Larios et al., 2009). An example item is: “*How often do you discipline your child for drinking a soda without your permission?”* Response options for each subscale were based on a 5-point Likert scale where 1 indicated “*Never*” or “*Disagree*,” and 5 indicated “*Always*” or “*Agree*.” Cronbach’s for limit setting, monitoring, discipline, control, and reinforcement were 0.93, 0.96, 0.92, 0.85, and 0.77, respectively. This measure was originally developed and validated in a sample of Hispanic parents, which demonstrated strong content, construct, and concurrent validity as well as high reliability in this population (Larios et al., 2009).

#### Adolescents’ Health-Related Outcome

##### Quality of Life

Quality of life (QOL; US Department of Health & Human Services, 2010) was measured using the sum of three adolescent-reported questions regarding the number of bad days they experience related to their physical and mental health for the past 30 days. The questions included: *“Now thinking about your physical health, which includes physical illness and injury, for how many days during the past 30 days was your physical health not good?*,*”* “*Now thinking about your mental health, which includes stress, depression, and problems with emotions, for how many days during the past 30 days was your mental health not good?*,*”* and *“During the past 30 days, for about how many days did poor physical or mental health keep you from doing your usual activities, such as self-care, work, or recreation?”.*

##### Anthropometric Measures

Participants’ height and weight were measured using a Seca 217 mobile stadiometer and a Seca 869 digital scale, respectively, by trained research staff. BMI percentiles were calculated using the Centers for Disease Control and Prevention (CDC) growth charts (Kuczmarski et al., 2002).

##### Physical Activity

Physical activity was assessed using the World Health Organization’s Global Physical Activity Questionnaire (GPAQ; Armstrong et al., 2018), a self-reported 7-day recall questionnaire with 36 items administered to adolescents. This questionnaire assesses physical activity duration (i.e., days, hours, minutes), intensity (i.e., moderate, vigorous), type (e.g., brisk walking, bicycling), and sedentary behaviors (e.g., video gaming). The items include physical activity during physical education class, lunch, immediately after school, in the evening, and on weekends, measured by the number of days/minutes of physical activity. In this study, we used a sum score of the total minutes of adolescents’ moderate to vigorous physical activity in a typical week.

##### Dietary Intake

The National Health and Nutrition Examination Survey Dietary Screener Questionnaire (DSQ; Thompson et al., 2017) was used to assess youth’s dietary intake. This 28-item self-reported measure assesses the consumption of 22 specific foods and drinks consumed over the past month in different settings including work, school, and restaurants. Responses were on a 9-point Likert scale and ranged from *never to 2 or more times per day*. Questions reflected both nutrient-dense options (e.g., whole fruits and vegetables) and calorie-dense options (e.g., doughnuts, soda, and cookies). The development and evaluation of this dietary questionnaire are described elsewhere (Thompson et al., 2017). Scoring algorithms were used to convert individuals’ daily intake into standard servings developed by the National Cancer Institute (available at: https://epi.grants.cancer.gov/nhanes/dietscreen/scoring/current/#scoring). Since the daily added sugar intake and sweetened beverages consumption were skewed positively, the results were log-transformed for analysis following George (2011).

##### Statistical Analysis

**Fig. 1:**
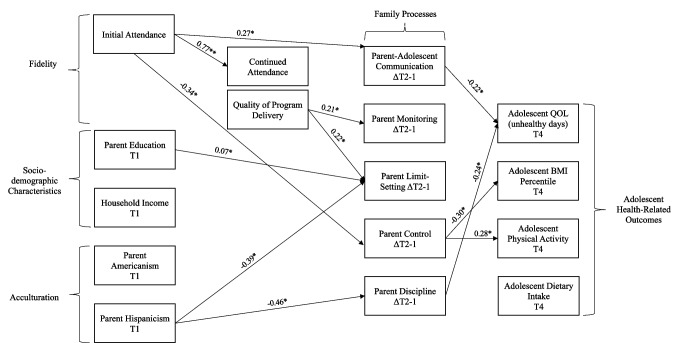
Study model. Note: the reported values are standardized path coefficients (not rounded). T1: baseline assessment; ΔT2-T1: the change between baseline assessment and 6 months after baseline assessment; T4: time four assessment, 24 months after baseline

Path analyses were used to test the hypothesized model (see Fig. 1). As depicted in the model, fidelity, socio-demographic characteristics, and acculturation were hypothesized to predict family processes, which in turn were hypothesized to impact adolescent health-related outcomes. We used data from baseline (T1), the changes in family processes scores between baseline and 6 months after baseline (ΔT2-1), as well as adolescent health-related outcomes at the 24 months post-baseline assessment. Additionally, a mediation analysis was used to assess whether family processes mediated the associations between fidelity, socio-demographic characteristics, acculturation, and adolescent health-related outcomes (MacKinnon, 2008). Using G-power software sample size calculations for regression analysis assumed a type I error = 0.05, a power = 0.80, and an effect size at a Cohen’s *d* = 0.20 (considered as a small effect). To meet this study’s hypothesis, a sample size of *n* = 98 participants was sufficiently powered, thus our sample size (*n* = 140) was adequate. Model fit was evaluated using two fit indices: (a) comparative fit index (CFI) ≥ 0.90 and (b) the root mean square error of approximation (RMSEA) ≤ 0.05 (Hu & Bentler, 1999). To handle missing data, full-information maximum likelihood (FIML) estimation was used (Enders, 2010). M-plus 8 for the analyses of the data (Muthén & Muthen, 2017).

## Results

### Hypothesized Model

Model fit indices of CFI = 0.96 and the RMSEA = 0.04 (90% CI = 0.01–0.09) suggest that the model is an adequate fit to the data. The study model is presented in Fig. 1. Results from the hypothesized paths are presented in the following sections. Means and standard deviations for initial and continued attendance as well as the quality of program delivery are presented in Table 2.

**Table 2:** Fidelity scores

**Fidelity Measures**	**mean (SD)**
Quality of program delivery	4.86 (0.21)
Initial attendance	2.37 (0.91)
Continued attendance	6.09 (3.19)

the possible ranges include quality of program delivery: 0–6; initial attendance: 0–3; continued attendance: 0–9

### Fidelity

Initial attendance was positively associated with continued attendance (*b* = 0.77, *p* < 0.001). Moreover, initial attendance was positively associated with changes (ΔT2-1) in parent-adolescent communication (*b* = 0.27, *p* = 0.05) and negatively associated with changes in parent control (*b* =  − 0.34, *p* = 0.01). The quality of program delivery was positively associated with changes in parental monitoring (*b* = 0.21, *p* = 0.02) and changes in parent limit setting (*b* = 0.22, *p* = 0.01). There were no other direct associations (*p* > 0.05).

### Acculturation and Demographic Characteristics

Parents’ education was positively associated with changes in parent limit-setting (*b* = 0.07, *p* = 0.013). Additionally, parents’ Hispanicism was positively associated with changes in parent discipline (*b* =  − 0.46, *p* = 0.002) and negatively associated with changes in parent limit-setting (*b* =  − 0.39, *p* = 0.008), such that higher parent Hispanicism was found to be associated with improvements in discipline and lower limit-setting.

### Adolescent Health-Related Outcomes

Quality of life was negatively associated with changes in parent-adolescent communication (*b* =  − 0.22, *p* = 0.03) and changes in parent discipline (*b* =  − 0.24, *p* = 0.016). In other words, improved parent-adolescent communication and improved parent discipline were significantly associated with a lower number of adolescent-reported unhealthy days. Changes in parent control were found to be negatively associated with adolescents’ BMI (*b* =  − 0.30, *p* = 0.009) and positively associated with adolescents’ physical activity (*b* = 0.28, *p* = 0.049). There was no significant association related to adolescents’ diet and food intake. Also, there were no significant direct associations between fidelity, socio-demographic characteristics, acculturation, and adolescent health-related outcomes (*p* > 0.05). Finally, the hypothesized mediational relationships were not significant.

## Discussion

This study evaluated the impact of dimensions of fidelity, individual-level socio-demographic characteristics, and acculturation on family-level processes and the impact of family-level processes on adolescent’s quality of life, BMI, physical activity, and dietary intake. The results show that: (1) quality of program delivery was positively associated with changes in parental monitoring and parent limit setting, (2) intervention attendance was positively associated with changes in parent-adolescent communication and negatively associated with changes in parent control, (3) parent education was positively associated with changes in parent limit setting, (4) Hispanicism was positively associated with changes in parent discipline and negatively associated with changes in parent limit setting, (5) changes in parent discipline and parent-adolescent communication were negatively associated with adolescents’ quality of life (i.e., fewer unhealthy days) and changes in parent control were associated with reduced adolescent BMI and increased physical activity. Our mediation analyses did not confirm our hypothesis that family-level processes would mediate the relationship between dimensions of fidelity, acculturation, and individual-level socio-demographic characteristics and adolescent health-related outcomes. Overall, it is important to consider multiple levels of influence when creating interventions related to health-related outcomes and behavioral changes. Our results demonstrated that in a family-based intervention that targets adolescents’ health, families’ sociodemographic characteristics, acculturation, and intervention fidelity by the study facilitators significantly affect parenting strategies, which in turn impacts their offspring’s health-related outcomes.

Quality of intervention delivery was associated with increases in monitoring and limit setting from baseline to post-intervention. The intervention activities included several messages related to healthy choices and gave the participants the opportunity to practice (role playing) what they were taught during the sessions. These activities, when delivered with enthusiasm and high quality, can enhance parents’ skills related to keeping track of what their adolescent is consuming and subsequently place limits on the amounts of energy-dense foods (e.g., soda) consumed by their adolescent.

Attendance was significantly associated with improvements in parent-adolescent communication and parent-controlling behaviors. Given that one of the first three group sessions of the intervention focused entirely on enhancing communication skills for health and wellness, it is not surprising that parents reported improvements in parent-adolescent communication. Parents may have also utilized these effective communication skills when having conversations with their child about the foods they are consuming. The effective communication skills in FUHW highlight the importance of validating and using positive reinforcement when communicating with adolescents as opposed to controlling styles of communication; parent-controlling behaviors may have therefore decreased in part due to the communication skills taught early in the intervention.

Related to acculturation, when foreign-born parents living in the USA retain their Hispanic cultural values and beliefs (i.e., Hispanicism), they are more likely to discipline their adolescent as it is related to diet and sedentary activity. This is likely due to the fact that immigrant mothers have reported challenges related to making healthy food choices for their families while maintaining traditional eating behaviors (Pineros-Leano et al., 2019). Ultimately, however, in the interest of protecting their family, Hispanic immigrant mothers are aware of the consequences of unhealthy eating and opt for healthier eating behaviors (Fernandez et al., 2022; Hammons et al., 2019; Moise et al., 2019), which may cause them to discipline adolescents if they do not comply with those behaviors. Hispanicism was negatively associated with changes in parent limit-setting, which is consistent with research that has shown that relative to White or Black children, Hispanic children have fewer limitations placed on them regarding the use of media (e.g., sedentary activity; Lindsay et al., 2018). In fact, Hispanic parents may even encourage and/or pressure their children to eat due to cultural beliefs that heavier children reflect good parenting and health. Conversely, parents’ education was found to be associated with changes in parent limit-setting. Previous research suggests that individuals with more education are four times more likely to comprehend nutrition labels than those with less than a high school degree (Sharif et al., 2014) and more likely to place limits on their child’s sedentary behaviors (Lehto et al., 2021; Määttä et al., 2017). This existing knowledge may have encouraged parents to place limits on energy-dense foods (e.g., foods with added sugar) and sedentary behaviors (e.g., time spent playing video games).

Changes in parent-adolescent communication and parent discipline were both negatively associated with quality of life, such that improvement in parent-adolescent communication and parental discipline led to adolescents reporting fewer unhealthy days related to their physical and mental health. This finding is consistent with that of other family-based healthy lifestyle interventions, which have also shown improvements in quality of life by improving family functioning (Fenner et al., 2016a, b). In this study, skills related to effective communication may have influenced the way that parents discipline their adolescents such that parents disciplined eating and activity behaviors in a context of understanding and support, which subsequently improved adolescents quality of life. Results also revealed that changes in parent control were negatively associated with BMI and positively associated with adolescent physical activity. Previous research has shown that controlling eating and activity practices have been associated with an increased risk for unhealthy eating among Latina adolescents (Arredondo et al., 2006; Power et al., 2015). However, given that adolescents in this study were adolescents with overweight and/or obesity, parents in this study may have utilized controlling behaviors as a response to manage obesogenic dietary intake and increase physical activity behaviors, subsequently resulting in lower BMI and increased physical activity (LeCroy et al., 2019).

### Implications

The findings of this study highlight the importance of examining multiple levels of influence (i.e., dimensions of fidelity, acculturation, individual-level sociodemographic characteristics, and family-level processes) and how they relate to Hispanic adolescent health-related outcomes. At the implementation level, dimensions of fidelity can help confirm that changes in outcomes are in fact attributable to a program (i.e., FUHW) if the program is delivered as intended (Dane & Schneider, 1998). Quality of delivery may not only impact continued attendance (i.e., dosage), but also simultaneously improve outcomes. In the FUHW intervention, quality of delivery assesses facilitators’ use of joining, group cohesion building, and participatory learning techniques in each of the intervention sessions, which if done correctly, may influence participants to continue engaging in the intervention. Intervention fidelity is not limited to what was measured in this study. Intervention fidelity also includes adherence, defined as whether the intervention is delivered as it was designed, and participant responsiveness, defined as the degree to which participants are involved in the intervention activities (Mihalic, 2004). Although using quality of program delivery and dosage as two important fidelity measures is extremely advantageous, adding additional dimensions including adherence and participant engagement provides researchers with a more cohesive and dynamic understanding of intervention fidelity and its impact on study outcomes.

Relatedly, parents may be drawn to the content presented early in the intervention. For Hispanic families in particular, eating is a social event that often occurs with family, and adolescents observe the eating behaviors and preferences of those around them (LeCroy et al., 2019; Williams et al., 2017). Given the salience of family for Hispanics, it is critical for family-based healthy lifestyle interventions to teach parents the skills and to facilitate productive conversations around healthy activity and eating behaviors in a context of support and understanding instead of communication that is controlling in nature and may subsequently disrupt family functioning and negatively impact diet and physical activity (Larios et al., 2009). Therefore, it may be prudent for family-based healthy lifestyle interventions to introduce topics such as effective communication early on in the program.

In this study, parent monitoring and parent limit setting did not impact adolescent health-related behaviors. Given that, there was a 21-month gap between the end of the intervention and the last follow-up period and changes in family-level processes occurred 3 months after the intervention. It may be that booster sessions focused on family-level processes and how they can be enhanced beyond the initial intervention and reiterate and problem-solve barriers to healthy eating and physical activity can help facilitate long-term effects on physical activity and dietary intake outcomes (Müller-Riemenschneider et al., 2008; Prado et al., 2020).

The findings from this study should be interpreted while considering some limitations. First, the Hispanic population in South Florida is not representative of the Hispanic population in the USA. Second, the sample size was modest and may have prevented our ability to detect significant relationships, in particular the mediation relationships. Third, the DSQ that was used for the participants diet provides a general understanding of dietary intake. Combining the DSQ with other measures such as 24-h recall will better capture some of the constructs of interest related to the participants’ dietary habits. Although there are limitations, this study has notable strengths: (1) the inclusion of determinants at multiple levels of influence (i.e., implementation, culture, socio-demographic, and family), (2) the longitudinal nature of the study and the inclusion of multiple assessments at several timepoints, and (3) the uniqueness of the study population.

## Conclusion

The findings of this study suggest that fidelity, parent education, and parent Hispanicism were related to changes in parent-adolescent communication and parenting strategies, which in turn impacted adolescent physical activity levels, BMI, and health-related quality of life at 21 months post-intervention. Our findings demonstrate the importance of focusing on implementation, acculturation, and family-level processes to improve obesity-related outcomes among an at-risk Hispanic population. Further research with larger sample sizes is required to examine the effect of intervention fidelity on health-related outcomes as well as the mediating effect of family processes on these relationships. Future implementation research can focus on the organizational (e.g., intervention location size) and environmental (e.g., ambiance) effects on the delivery of the interventions to improve the study outcomes.
