# Correction: The Role of Intervention Fidelity, Culture, and Individual-Level Factors on Health-Related Outcomes Among Hispanic Adolescents with Unhealthy Weight: Findings from a Longitudinal Intervention Trial

**DOI:** 10.1007/s11121-024-01666-x

**Published:** 2024-03-20

**Authors:** Padideh Lovan, Alyssa Lozano, Yannine Estrada, Cynthia Lebron, Tae Kyoung Lee, Sarah E. Messiah, Guillermo Prado

**Affiliations:** 1 Department of Psychology, University of Miami, Miami, FL USA; 2 Sylvester Comprehensive Cancer Center, University of Miami, Miller School of Medicine, Miami, FL USA; 3 Department of Public Health Sciences, University of Miami Miller School of Medicine, Miami, FL USA; 4 School of Nursing and Health Studies, University of Miami, Miami, FL USA; 5 Department of Child Psychology and Education, Sungkyunkwan University, Seoul, South Korea; 6 Department of Epidemiology, School of Public Health, Human Genetics and Environmental Science, The University of Texas Health Science Center at Houston, Dallas, TX USA; 7 Center for Pediatric Population Health, School of Public Health, University of Texas Health Science Center, Dallas, TX USA; 8 Department of Pediatrics, McGovern Medical School, Houston, TX USA

**Correction to: Prevention Science** 10.1007/s11121-023-01527-z

The article “The Role of Intervention Fidelity, Culture, and Individual‑Level Factors on Health‑Related Outcomes Among Hispanic Adolescents with Unhealthy Weight: Findings from a Longitudinal Intervention Trial”, written by Lovan, P., Lozano, A., Estrada, Y., Lebron, C., Lee, T.K., Messiah, S.E., and Prado, G., was originally published electronically on the publisher’s internet portal on 18 April 2023 without open access. With the author(s)’ decision to opt for Open Choice the copyright of the article changed on 12 December 2023 to © The Author(s) 2023 and the article is forthwith distributed under a Creative Commons Attribution 4.0 International License, which permits use, sharing, adaptation, distribution and reproduction in any medium or format, as long as you give appropriate credit to the original author(s) and the source, provide a link to the Creative Commons licence, and indicate if changes were made. The images or other third party material in this article are included in the article’s Creative Commons licence, unless indicated otherwise in a credit line to the material. If material is not included in the article’s Creative Commons licence and your intended use is not permitted by statutory regulation or exceeds the permitted use, you will need to obtain permission directly from the copyright holder. To view a copy of this licence, visit http://creativecommons.org/licenses/by/4.0/.

The original article has been corrected.

